# Recombinant horse interleukin-4 and interleukin-10 induced a mixed inflammatory cytokine response in horse peripheral blood mononuclear cells

**DOI:** 10.14202/vetworld.2019.496-503

**Published:** 2019-04-03

**Authors:** Sheetal Saini, Harisankar Singha, Priyanka Siwach, B. N. Tripathi

**Affiliations:** 1 ICAR-National Research Centre on Equines, Hisar, Haryana, India; 2 Department of Biotechnology, Chaudhary Devi Lal University, Sirsa, Haryana, India

**Keywords:** cell proliferation, cytokines, enzyme-linked immunosorbent assay, real-time polymerase chain reaction

## Abstract

**Background and Aim:**

Interleukin (IL)-4 and IL-10 activate plethora of immune cells and induce the humoral immune response. However, recombinant version of horse IL-4 and IL-10 has not been investigated to understand their immunomodulating activities. This study aimed to produce recombinant horse mature IL-4 and IL-10 in *Escherichia coli*. Immune-modulating activities of recombinant horse IL-4 and IL-10 were investigated in peripheral blood mononuclear cells (PBMCs).

**Materials and Methods:**

Equine PBMCs were stimulated with recombinant IL-4 and IL-10. A proliferation of PBMCs was measured by XTT assay and cytokines induction was measured by enzyme-linked immunosorbent assay and real-time polymerase chain reaction.

**Results:**

Sodium dodecyl sulfate-polyacrylamide gel electrophoresis analysis displayed a molecular weight of 15 kDa for IL-4 and 19 kDa for IL-10. Recombinant IL-4 and IL-10 significantly induced cell proliferation at 250 ng/ml. The results demonstrated that IL-4 enhanced expression of interferon-gamma (IFN-γ), IL-6, tumor necrosis factor-alpha (TNF-α), and IL-10, while recombinant horse IL-10 induced expression of IL-6, IFN-γ, and TNF-α.

**Conclusion:**

The present study demonstrated that biologically active horse IL-4 and IL-10 could be produced in *E. coli*.

## Introduction

Interleukin (IL)-4 and IL-10 are considered as important Th2 cytokines that promote growth and differentiation for a variety of immune cells such as T cells, NK cells, B cells [1,2], and augments antibody-mediated immune response against extracellular bacteria and parasites. IL-4 promotes growth and differentiation of B-cells, tissue repair, maintains allergic responses and homeostasis [3-5]. IL-10 is a multifunctional cytokine produced by a variety of cell types including Th2 cells, dendritic cells, activated macrophages, B cells, and mast cells [6,7]. Its suppressive effects on dendritic cells and macrophages cause inhibition of the production of pro-inflammatory cytokines, IL-12 and decrease the development of Th1 cells [6].

Assessment of cytokine profile provides clinical insights in understanding host responses to infectious as well as autoimmune diseases. IL-4 and IL-10 have been shown to play an important role in the regulation of IgE-mediated allergic reaction such as recurrent airway obstruction or heaves, uveitis, and insect bite hypersensitivity in equines [8-10]. However, very few studies were conducted either to decipher the role of these cytokines in response to equine bacterial and viral infection or to assess the immunomodulatory effect of recombinant IL-4 and IL-10. Earlier, horse interleukin (IL)-4 and granulocyte-macrophage-colony-stimulating factor (GM-CSF) expressed in a mammalian expression system were bioactive in the lymphocyte costimulatory system and on equine monocyte [11]. One study reported that recombinant equine IL-4 expressed in Chinese hamster ovary cells was able to induce lymphocyte proliferation in leukoagglutinin prestimulated equine peripheral blood mononuclear cells (PBMCs) [12]. Wagner *et al*. reported expression of equine cytokines (IL-2, IL-4, and transforming growth factor-β1) fusion with IgG1 heavy chain constant region as a tag for detection of antibody specificity to equine cytokine [13]. A very recent report showed that priming with interferon gamma (IFN-γ), tumor necrosis factor-alpha (TNF-α), or IL-6 significantly decreased intracellular replication of *Rhodococcus equi* in equine macrophages, but IL-10 or IL-1β increased intracellular survival of *R. equi* [14]. Neonatal immunization with equine herpesvirus type 1 (EHV-I) glycoprotein C and IL-4 mediated improved antibody response and partial protection against EHV-1 challenge [15]. However, no published report is available on expression, purification of IL-10, and its utility as adjuvant or therapeutic effect of horse IL-10.

The present study aimed to produce recombinant horse IL-4 and IL-10 in a prokaryotic expression system and investigate immuno-modulating effect of these cytokines in horse PBMCs.

## Materials and Methods

### Ethical approval

Blood samples from six Marwari horses of either sex between 3 and 4 years old were collected at a different time point of experiments carried out from 2014 to 2016. Collection of blood samples from horses was approved by the Institute Animal Ethics Committee vide NRCE/CPCSEA/2013-2014. Horses were kept at animal shed NRCE, Hisar. 10 ml blood samples were aseptically collected from jugular vein using 18 gauge needles in ethylenediaminetetraacetic acid for isolation of PBMC.

### Cloning and expression of horse IL-4 and IL-10

Coding sequences of Marwari horse mature IL-4 and IL-10 gene [16] were subcloned into *Sac* I and *Hind* III (Thermo Scientific FastDigest, UK) restricted prokaryotic expression vector pQE30 (Qiagen, Germany). The recombinant pQE30 vectors with respective IL-4 and IL-10 insert were transformed in *Escherichia coli* M15 competent cells prepared by Z-competent™ *E. coli* transformation kit (Zymo Research, USA). Positive clones were selected on Luria Bertani agar (LBA) plates supplemented with kanamycin (30 μg/ml) and ampicillin (50 μg/ml) (HiMedia, India) and screened by sodium dodecyl sulfate-polyacrylamide gel electrophoresis (SDS-PAGE). Briefly, 10 colonies were randomly picked and were grown in 5 ml of LB broth at 37°C at 170 rpm. At mid-log phase (approximate O.D_600_ 0.6), 1 mM isopropyl-β-D-thiogalactoside (IPTG) (Promega, USA) was added to culture and was further incubated for 3-6 h. Samples from bacterial cells were prepared as per standard protocol [17] and run on 12% SDS-PAGE. The gel was stained by Coomassie brilliant blue dye and protein band was visualized by destaining. For further confirmation of insert, two positive recombinant clones were sequenced by Sanger method (Eurofins genomics, India) and sequences were verified by NCBI-BLAST (https://blast.ncbi.nlm.nih.gov/Blast.cgi) program.

### Purification of recombinant IL-4 and IL-10

For purification, recombinant IL-4 and IL-10 positive clones were grown in LB broth (300 ml) for IPTG induction. Bacterial cells were harvested by centrifugation at 8000 rpm for 15 min. Pellet was resuspended in 50 ml of lysis buffer (100 mM sodium phosphate, 10 mM Tris-HCl, and 8 M urea pH 8.0) plus 1 mg/ml lysozyme (Sigma, USA) and incubated on ice for 30 min. Cell suspensions were sonicated at 200 W for 6 times (10 s pulse and 10 s pause). Cell lysate was centrifuged at 12,000 rpm for 20 min at 4°C, and recombinant cytokines were purified from clear supernatant by nickel-nitrilotriacetic (Ni-NTA) resin following manufacturer’s protocol (Qiagen, Germany). Different fractions were collected during the purification process and run on 12% SDS-PAGE. Similarly, sham purification eluates were also prepared from *E. coli* cells harboring empty pQE30 vector. The eluted fractions containing purified recombinant IL-4 and IL-10 were pooled together and dialyzed at 4°C using Slide-A-Lyzer^®^ Dialysis Cassettes (Thermo Scientific, USA) in phosphate-buffered saline (PBS) and were quantified by the Bradford method (1976). Purified recombinant IL-4 and IL-10 were stored in aliquots at −20°C for further use.

### PBMCs isolation and stimulation with recombinant IL-4 and IL-10

Horse PBMCs were isolated as per previous protocol [16]. Briefly, PBMCs were seeded at a density of 2×10^5^ per well in RPMI 1640-1X medium (GIBCO Life Technologies^™^, UK) supplemented with 10% fetal calf serum (Sigma, USA), 100 U/ml penicillin, and 100 U/ml streptomycin. Different concentrations of recombinant IL-4 and IL-10 ranging from 250 to 1500 ng/ml (at 250 ng increment) were used for the stimulation of PBMCs. The PBMCs were incubated at 37°C in humid atmosphere of 5% CO_2_ tension. Culture plates were regularly monitored under microscope till harvesting. Cells were harvested at defined time interval for different assays such as 72 h for lymphocyte proliferation, 48 h for cytokine enzyme-linked immunosorbent assay (ELISA), and 3 h and 24 h for real-time polymerase chain reaction (PCR). Concanavalin A (5 µg/ml) (Sigma, USA) and phytohemagglutinin-A (10 µg/ml) (Sigma, USA) were used as positive control mitogen. PBS-stimulated equine PBMCs were used as negative control. Sham control eluates were used for ascertaining residual proliferative effect of purified fractions obtained during purification process.

### Lymphocyte proliferation assay by XTT

A proliferation of PBMCs was measured by XTT assay after 72 h of post-stimulation. The XTT working solution was prepared by adding 10 mg of XTT reagent (Biotium, USA) in 10 ml of RPMI media without phenol red (Sigma, USA) plus 100 μl of phenazine methosulfate (PMS, Sigma, USA). In culture plate, 50 μl of XTT solution was added in triplicate wells. Plates were shaken gently for mixing of dye and incubated at 37°C for 4 h. The absorbance was measured at 500 nm and at 600 nm for reference. Stimulation index (SI) was calculated as the ratio between optical density (OD) values of stimulated cells to unstimulated cells.


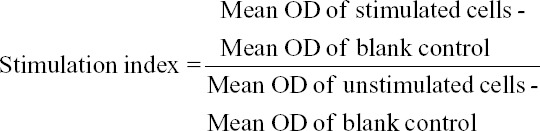


### Quantification of IL-4, IL-10, IFN-γ, and TNF-α in PBMCs culture supernatant by ELISA

After 48 h of post-stimulation, cell culture supernatants from each well were harvested, cellular debris was separated by centrifugation, and clear supernatant was kept at −80°C for measurement of cytokines by ELISA. For estimation of IL-10 and IFN-γ, sandwich ELISA kit was used according to manufacturer’s instructions (Ray Bio^®^, USA). For IL-4 and TNF-α, a competitive ELISA kit was used (BlueGene, Shanghai) as per manufacturer’s instructions. Two-fold dilutions of respective standard cytokines (0–500 pg/ml) were added in triplicate wells in 100 μl volume. Similarly, 100 μl culture supernatants were added in triplicate wells. Finally, OD was measured at 450 nm in ELISA plate reader (Multiskan Go, Thermo Scientific, Finland). The standard curve of each reference cytokine was plotted, and cytokines under investigation in culture supernatants were quantified from the standard curve.

### Quantification of cytokine mRNA by real-time quantitative PCR (qPCR)

#### Preparation of standard curves

Primers used for quantitative real-time PCR for equine IL-2, IL-4, IL-10, IL-18, IFN-γ, IL-6, TNF-α, IL-12p35, and housekeeping gene ß-actin are shown in Supplementary Table-1. Primer sequences against TNF-α, IL-6 [18], and IL-12 [19] genes were taken from the previous studies. Five-fold serial dilutions of known copy number recombinant cytokines clones were used for standard curve preparation as previously described method [20]. The clone of respective cytokine gene in pGEM-T Easy vector was purified with nucleospin plasmid mini kit (Nucleospin, Germany) and nucleic acid concentration was determined by Nanodrop spectrophotometer (Eppendorf, Biophotometer Plus Spectrophotometer, Germany). The number of copies/µl was calculated according to the following formula:

**Supplementary Table-1:** Primer sets for SYBR Green qRT-PCR, target cytokine, and standard curve data.

Target gene	Primer sequence (5’- 3’)	References	Length (bp)	E%	Slope	RSq	S	Inter-assay CV%	Intra-assay CV%
IL-2	F: CCAAGAAGGCCACAGAATTG R: GACCCCTTTAGTCCCAGAAC	This study	145	96.5	−3.41	0.995	10	4.04	2.72
IL-4	F: TGGCCCGAAGAACACAGATG R: CTTGAGGTTCCTGTCCAGTCC	This study	124	108	−3.14	0.995	10^2^	10.5	1.51
IL-10	F: TCTGCCCTGTGAAAATAAGAGC R: GTCAAACTCACTCATGGCTTTG	This study	100	105	−3.19	0.975	10^2^	7.39	0.74
IL-18	F: TCTAGCGGTAACCATCTCTGTG R: GTCCTGGAACACTTCTCTGAAAG	This study	149	101.5	−3.29	0.991	10	2.97	2.30
IFN-g	F: GGAGGACCTGTTCGTTAAGTTC R: TGGGCGACAGATCATTCATC	This study	146	106.2	−3.17	0.987	10	3.05	0.35
IL-6	F: CACCGAGCTCACCCCACTACC R: CTACATTATCCGAACAG	[18]	123	110.18	−3.1	0.997	10	2.47	0.63
TNF-a	F: CACCAGCACTGAAAGCATGATC R: TCACAGGGCAATGATCCCAAAG	[18]	134	101.91	−3.28	0.974	10	5.09	1.58
IL-12p35	F: CCAGACGCTGTGCCTTAGC R: TCTGCCTCTGAGGATCTATCAACA	[19]	140	106.54	−3.17	0.987	10	2.18	0.59
b-actin	F: ACAGGATGCAGAAGGAGATCAC R: TGCTGGAAGGTGGACAATGAG	This study	130	101	−3.25	0.991	10	4.23	0.72

F=Forward, R=Reverse, E=Reaction efficiency, RSq=Linear correlation, S=Limit of sensitivity, bp=Base pair, CV=Coefficient of variance. qRT-PCR=Quantitative reverse transcription polymerase chain reaction


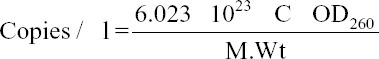


Where,

C = Concentration of plasmid DNA in g/µl and M.Wt = Molecular weight of cytokine plasmid (base pair 660× *g*).

PCR reaction consisted of 5 µl of 2× SYBR Green master mix (Clontech, USA), 1 µl of each primer (2 μM), and 1 µl of plasmid DNA in a final volume of 10 µl. A non-template control (RNase-free water) was included for every PCR run. Thermal program was set at 95°C for 3 min for initial denaturation and 40 cycles of 95°C for 5 s and 60°C for 30 s and ran on CFX96^™^ real-time system (Bio-Rad, Singapore). A melting curve analysis was performed after the amplification phase to determine any non-specific amplification or primer-dimer formation. Repeatability and reproducibility of the qPCR assays were determined by intra-assay and interassay variation, respectively [18].

#### RNA isolation, cDNA synthesis, and real-time PCR

PBMCs were harvested at 3 h and 24 h post-stimulation and RNA was isolated using Tri-reagent (Sigma, USA) according to the manufacturer’s instructions. The purity and quantity of RNA were assessed using a NanoDrop spectrophotometer (Eppendorf, Biophotometer Plus Spectrophotometer, Germany). Subsequently, 1 μg of RNA was reverse transcribed using SmartScribe^™^ reverse transcriptase cDNA kit (Clontech, USA). Following synthesis, cDNA was stored at −20°C. Real-time quantitative reverse transcription-PCR was performed in 10 µl volume including 5 µl of a 2× SYBR Green master mix (Clontech, USA), 1 µl of each primer (2 μM), and 1 µl of cDNA and nuclease-free water. A non-template control (RNase-free water) and no RT control were included for every PCR run. All reactions were performed in duplicates, and same thermal conditions were followed as mentioned above. The specificity and size of real-time PCR products for each cytokine were confirmed by 2% agarose gel electrophoresis and sequencing of PCR products. Copy number of cytokines in control and stimulated PBMCs was calculated by extrapolating the C_t_ value to the standard curves.

### Statistical analysis

Data were analyzed using the GraphPad Prism program, version 5.01 (GraphPad Prism Software, San Diego, CA, USA). Statistical significance for XTT assay and cytokine ELISA was evaluated by one-way analysis of variance (ANOVA) followed by Turkey’s multiple comparison tests. Significant differences among cytokines in terms of copy numbers were evaluated by two-way ANOVA. Results were expressed as the mean ± standard error mean. All experiments were repeated at least 3 times to ensure reproducibility. Data were considered significant when p<0.05.

## Results

### Expression and purification of IL-4 and IL-10

**Figure-1:**
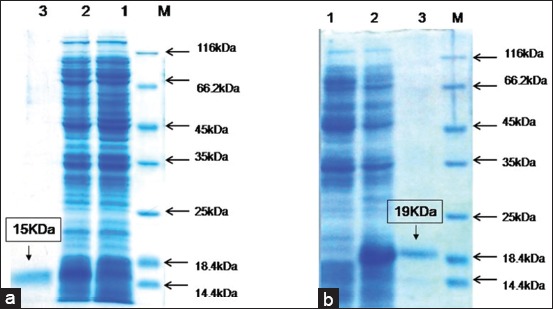
SDS-PAGE analysis showing expression of 15 kDa recombinant IL-4 (a) and 19 kDa recombinant IL-10 (b) in M15 cells: Lane 1-Uninduced control; Lane 2-recombinant IL-4 and IL-10 in IPTG-induced cells; Lane 3: Purified recombinant IL-4 (a) and IL-10 (b); Lane M, protein molecular weight marker (ranging from 14.4 kDa to 116 kDa).

Optimum expression of recombinant horse IL-4 and IL-10 were observed at 4 h after IPTG induction. For purification, optimum binding time with Ni-NTA resin was 6 h. SDS-PAGE analysis showed that molecular weight of the mature recombinant IL-4 and IL-10 was 15 kDa and 19 kDa, respectively (Figure-1a and b). No copurification of *E. coli* protein was detected in recombinant IL-4 and IL-10 eluates.

### Lymphocyte proliferation assay

**Figure-2:**
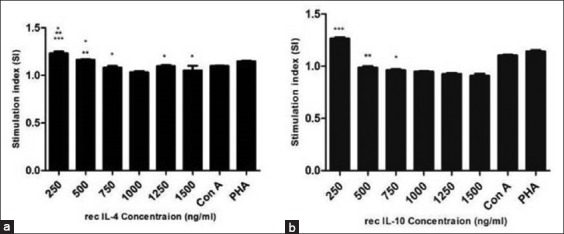
Results of proliferation assay were expressed as the mean stimulation index±SEM (standard error mean) obtained from three independent experiments. Symbol of *^,^ **^,^ and *** indicating p-value having significant difference p<0.05, p<0.01, and p<0.001, respectively. Briefly, horses peripheral blood mononuclear cells (2×10^5^/well) were stimulated with different concentrations (250-1500 ng/ml) of recombinant interleukin (IL)-4 (a) and IL-10 (b), and cellular proliferation was measured after 72 h.

Growth inducing and cellular proliferative effect (SI) at a different dose of recombinant IL-4 and IL-10 are shown in Figure-2a and b, respectively. At 250 ng/ml concentration, recombinant (rec) IL-4 and IL-10 had a significant effect on proliferation of equine PBMCs (p<0.001, SI ~1.3). Con A and PHA induced proliferative effect on equine PBMCs (SI ~1.1). Comparison of the highest SI obtained by recombinant cytokines with mitogens (Con A and PHA) was highly significant (p<0.001).

### Measurements of IL-4, IL-10, IFN-γ, and TNF-α in PBMCs culture supernatant by ELISA

At 500 ng/ml concentration, recombinant IL-4 significantly induced secretion of IL-10 (278.7 pg/ml, p<0.001) and TNF-α (196.6 pg/ml, p<0.001) (Table-1). Recombinant IL-10 induced secretion of TNF-α (201.10 pg/ml), IFN-γ (173.7 pg/ml), and IL-4 (134.1 pg/ml). On the other hand, Con A and PHA significantly enhanced secretion of IL-4, IL-10, TNF-α, and IFN-γ (p<0.001) as compared to PBS and sham control-stimulated PBMCs. Comparative analysis revealed that recombinant IL-4, IL-10, Con A, and PHA were equally potent stimulator for induction of TNF-α (Table-1). Recombinant IL-4 had maximum stimulatory effect on IL-10 production but had least effect on IFN-γ production.

**Table-1:** Quantification of IL-4, IL-10, IFN-γ, and TNF-α cytokine in PBMCs culture supernatants by ELISA.

Cytokines in culture supernatants	Rec-IL-4 (500 ng/ml)	Rec-IL-10 (500 ng/ml)	Con A	PHA	Control	Sham control
IL-4 (pg/ml)	NT	134.1±1.15	212.6±3.4^#^	144.6±2.49^#^	31.65±1.04	39.15±2.35
IL-10 (pg/ml)	278.7±4.50^#^	NT	113.8±2.58^#^	134.0±2.50^#^	36.10±1.14	27.60±1.60
TNF-α (pg/ml)	196.6±2.50^#^	201.10±2.22^#^	194.6±2.45^#^	198.9±2.32^#^	32.22±1.23	36.35±2.66
IFN-γ (pg/ml)	65.68±3.55	173.7±4.75^#^	113.4±2.38^#^	135.0±2.26^#^	36.45±1.10	35.89±2.34

Results obtained at 500 ng/ml concentration of recombinant IL-4 and IL-10 are shown here. Values were expressed as mean±SEM.

# Indicating p-value having significant difference p<0.001. NT=Not tested, IL=Interleukin, IFN-γ=Interferon gamma. TNF-α=Tumor necrosis factor-alpha, PBMCs=Peripheral blood mononuclear cells, ELISA=Enzyme-linked immunosorbent assay

### Quantification of cytokine mRNA in recombinant IL-4 and IL-10 stimulated PBMCs by real-time RT-PCR

**Figure-3:**
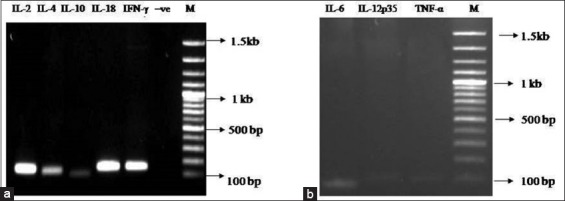
Agarose gel electrophoresis of cytokine fragment of IL-2, IL-4, IL-10, IL-18, IFN-γ, -ve control (Figure-3a) and IL-6, IL-12p35,TNF-α (Figure-3b) obtained by real-time PCR. Lane M: Molecular size DNA ladder (100bp).

Characteristic statistical value of real-time PCR standard curves such as slope, correlation coefficient, efficiency, and sensitivity of each cytokine is shown in supplementary Table-1. The correlation coefficients of the standard curves ranged from 0.975 to 0.997. Melting curve analysis showed a single sharp peak, indicating specific amplification. All RT-PCR amplicons were of predicted size ranging from 100 to 149 bp (Figure-3). DNA sequencing of PCR products and subsequent BLAST analysis confirmed identity of targeted cytokine fragment (data not shown).

**Figure-4:**
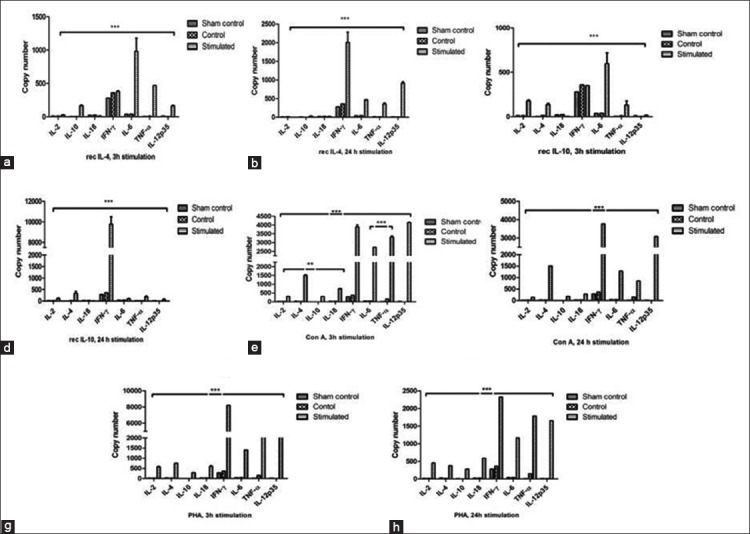
(a-h) Quantification of equine cytokines in horse peripheral blood mononuclear cells. Quantification of cytokine mRNA in horse PBMCs by real-time PCR at 500 ng/ml concentration of recombinant IL-4 (Figure-4a and b), IL-10 (Figure-4c and d), mitogens Con A (Figure-4e and f), and PHA (Figure-4g and h) at 3 h and 24 h post-stimulation. Results represent the mean copy number of cytokines±SEM. Symbol of ** and *** indicating p-value having significant difference at p<0.01 nd 0.001, respectively.

Copy numbers (Cn) of horse cytokine mRNA investigated in this study are shown in Figure-4. Recombinant IL-4-treated PBMCs showed predominant expression of IL-6 (Cn=979.4) and TNF-α (Cn=467.1) at 3 h (Figure-4a). However, significant upregulation of IFN-γ (Cn=2007) and IL-12p35 (Cn=911) mRNA was observed (p<0.001) (Figure-4b) at 24 h. In contrast, recombinant IL-4 had no significant effect on IL-2, IL-10, and IL-18 mRNA expression.

In PBMCs stimulated with recombinant IL-10, significant upregulation of IL-6 (Cn=596.9, p<0.001) and IFN-γ (Cn= 9762, p<0.001) mRNA was observed at 3 h and 24 h, respectively (Figure-4c and d). However, recombinant IL-10 had minimal effect on IL-2, TNF-α, and IL-4 expression. Con A induced significant upregulation for IL-12p35, IFN-γ, TNF-α, and IL-6 (Figure-4e and f) at 3h and 24 h, respectively. PHA significantly enhanced mRNA expression of IFN-γ and IL-12p35 TNF-α (Figure-4g and h) at 3h and 24 h post-stimulation.

## Discussion

In the present study, recombinant horse IL-4 and IL-10 were produced in prokaryotic expression system, and immuno-modulating activities of these two cytokines were studied in horse PBMCs. Recombinant IL-4 and IL-10 markedly enhanced growth and proliferation of horse PBMCs. Besides, recombinant horse IL-4 significantly induced secretion of IL-10 and TNF-α in PBMCs culture supernatants and upregulated IFN-γ, IL-6, and IL-12p35 mRNA expression. It was suggested that IL-4 activates c-Maf transcription factor for *in vivo* expression of IL-10 [21]. One of the recent studies showed that IL-4 enhances IL-10 production by Th1 cells and ameliorate Th1 driven pathology in infectious, allergic, and autoimmune diseases [1]. Similarly, induction of IFN-γ expression by mouse recombinant IL-4 was also reported by Morris *et al*. [22]. The current findings suggested that recombinant IL-4 significantly induced mRNA transcription of IL-6 at 3 h and IFN-γ and IL-12p35 at 24 h. The time-dependent response of horse PBMCs regarding IFN-γ expression observed in the present study is comparable to the previous findings, where mouse IL-4 induced IFN-γ production by NK and NKT cells to a less extent at 3 h and to a greater extent after 24 h stimulation [22]. Oliver *et al*. showed that murine IL-4 was able to potentially stimulate IFN-γ in a STAT6 independent manner even in the absence of T cell receptor in murine CD8+ T cells [23]. In another study, recombinant equine IL-4 induced a mixed inflammatory response in equine peripheral blood neutrophils with increased expression of IL-8 and TNF-α [24]. Significant upregulation of IL-12p35 by recombinant horse IL-4 can be supported by earlier findings where, IL-4 indirectly, lead Th1 cell differentiation to enhance the production and bioactivity of IL-12 in mouse and human dendritic cells [25]. In consensus with the previous findings, recombinant horse IL-4 in the present study showed immunostimulatory effect on PBMCs and induced Th1 cytokines (IFN-γ and IL-12p35), Th2 cytokines (IL-10), and pro-inflammatory cytokines (IL-6 and TNF-α).

IL-10 is an anti-inflammatory cytokine that plays a crucial role in preventing inflammatory and autoimmune pathogenesis and immune homeostasis [6,26,27]. Here, we have demonstrated that recombinant horse IL-10 significantly enhanced production of TNF-α, IFN-γ, and IL-4 in PBMCs culture supernatant and upregulated IL-6 and IFN-γ mRNA at 3 h and 24 h post-stimulation, respectively. In comparison to IL-4, recombinant IL-10 had more profound effect on upregulation of IFN-γ (p<0.001, IL-10 vs. IL-4) at 24 h. However, it had very weak effect in induction of IL-18, IL-4, IL-2, and IL-12p35 mRNA. The previous studies have reported that human and murine IL-10 potently inhibits production of IL-6, IL-10 itself, IFN-γ, IL-12, IL-18, GM-CSF, G-CSF, M-CSF, TNF-α, LIF, and PAF by activated monocytes/macrophages [28,29]. However, in the present study, contrast findings were observed. Recombinant horse IL-10 significantly elevated production of IFN-γ, TNF-α, and IL-4 by horse PBMCs. However, mechanism by which recombinant horse IL-10 exerts such high-level induction of IFN-γ remains unexplained and warrants future research.

The mitogens (Con A and PHA) elevated the mRNA expression of TNF-α, IFN-γ, IL-2, IL-4, IL-12p35, and IL-6. In real-time PCR, significant induction of cytokine mRNA was observed at 3 h and 24 h post-stimulation and transcripts were barely detected after 48 h and 72 h. The previous studies also suggested optimum expression of TNF-α and IFN-γ following 3-6 h stimulation of human PBMCs by PHA [30] and TNF-α, IFN-γ, IL-2, IL-4, and IL-10 in Con A stimulated rabbit splenocytes [31]. This may be due to rapid decay of cytokine mRNA which is related to interaction between AU-rich cis-elements in 3 UTR and trans-acting RNA binding proteins [32]. Similar observation obtained in the present study using Con A and PHA further validates the previous findings in equine PBMCs.

## Conclusion

Findings of the present study suggested that prokaryotic expression system can be used for the production of biologically active recombinant horse IL-4 and IL-10. Furthermore, recombinant horse IL-4 and IL-10 induced a mixed inflammatory cytokine profile in horse PBMCs. The future experiments may be designed to determine the immune modulating activity of recombinant horse IL-4 and IL-10 in equine disease model.

## Authors’ Contributions

SS executed research experiments, data analysis, and drafted the manuscript. HS designed the project and technical programme, supervised the work and data analysis, and edited the manuscript. PS guided to SS and helped in revision and editing of the manuscript. BNT supervised the project activities and edited the manuscript. All authors read and approved the final manuscript.
